# Prevalence of uncontrolled hypertension and contributing factors in Ethiopia: a systematic review and meta-analysis

**DOI:** 10.3389/fcvm.2024.1335823

**Published:** 2024-04-09

**Authors:** Mengistie Yirsaw Gobezie, Minimize Hassen, Nuhamin Alemayehu Tesfaye, Tewodros Solomon, Mulat Belete Demessie, Teklehaimanot Fentie Wendie, Getachew Tadesse, Tesfaye Dessale Kassa, Fentaw Tadese Berhe

**Affiliations:** ^1^ Department of Clinical Pharmacy, School of Pharmacy, College of Medicine and Health Sciences, Wollo University, Dessie, Ethiopia; ^2^ Department of Statistics, College of Natural Sciences, Wollo University, Dessie, Ethiopia; ^3^ Department of Clinical Pharmacy, College of Health Sciences, Mekelle University, Mekelle, Ethiopia; ^4^ Department of Epidemiology and Biostatistics, School of Public Health, College of Medicine and Health Sciences, Wollo University, Dessie, Ethiopia; ^5^ Public Health & Economics Modeling Group, School of Medicine & Dentistry, Griffith University, Gold Coast, QLD, Australia

**Keywords:** hypertension, cardiovascular disease, systematic review, meta-analysis, Ethiopia

## Abstract

**Background:**

Uncontrolled hypertension (HTN) is a major risk factor for cardiovascular and cerebrovascular disease. The prevalence of HTN in the Ethiopian adult population is almost 20%.This study aimed to determine the prevalence of uncontrolled HTN and its contributing factors among patients with HTN in Ethiopia undergoing treatment.

**Methods:**

Electronic bibliographic databases such as PubMed, Google Scholar, Hinari (Research4Life), Embase, and Scopus were searched for original records in the English language that assessed HTN control in Ethiopia and were available before 29 June 2023. The data were extracted using a format prepared in Microsoft Excel and exported to the software STATA 17.0 for analysis. The study protocol was registered at PROSPERO with the reference number CRD42023440121.

**Results:**

A total of 26 studies with 9,046 patients with HTN were included in the systematic review and meta-analysis, of which 11 studies were used to assess factors contributing to uncontrolled blood pressure (BP) in patients in Ethiopia. The estimated prevalence of uncontrolled HTN in the population of Ethiopia is 51% [95% confidence interval (CI), 42%–60%]. The subgroup analysis, based on the assessment tools, region, and follow-up period, revealed that the prevalence of uncontrolled BP was highest following the guidelines of the American Heart Association/American College of Cardiology (AHA/ACC) (89%; 95% CI: 87%–91%) and in Addis Ababa (58%; 95% CI: 40%–76%), and the lowest proportion of uncontrolled BP was in the 3-month follow-up period (34%; 95% CI: 29%–39%). The presence of diabetes mellitus showed the highest impact (pooled odds ratio: 5.19; CI: 1.41–19.11) for uncontrolled HTN. The univariate meta-regression method confirmed that the sample size, year of publication, and subgroups were not sources of heterogeneity in the pooled estimates. Egger's regression test did not indicate the presence of publication bias.

**Conclusion:**

More than half of the hypertensive patients in Ethiopia have uncontrolled BP. Diabetes mellitus, advanced age, male sex, and the presence of comorbidities are among the factors contributing to uncontrolled HTN in Ethiopia. The concerned bodies working in this area should implement interventional strategies and recommendations that might be helpful in achieving optimal BP in hypertensive patients.

**Systematic Review Registration:**

https://www.crd.york.ac.uk/prospero/display_record.php?ID=CRD42023440121, PROSPERO (CRD42023440121).

## Introduction

Hypertension (HTN) is a common disease characterized by a persistently elevated arterial blood pressure (BP). According to the 2017 American Heart Association/American College of Cardiology (AHA/ACC) Blood Pressure Guideline, HTN is defined as a BP of ≥140/90 to ≥130/80 mmHg based on an average of ≥2 readings taken at ≥2 visits. Thus, awareness and treatment in adults are based on the systolic BP (SBP)/diastolic BP (DBP) cutoff points of 130/80 mmHg, and control is based on an SBP/DBP <130/80 mmHg (1).

According to the 2014 reports of the World Health Organization (WHO), the global prevalence of raised BP in adults is approximately 22% (2). In sub-Saharan Africa (SSA), an estimated 74.7 million individuals are hypertensive, and by the year 2025, the number of hypertensive individuals is projected to increase by 68% to 125.5 million individuals (3). A recent meta-analysis study in Ethiopia revealed that the prevalence of HTN among the Ethiopian adult population is almost 20% (4). This finding indicates that HTN is a public health concern in Ethiopia.

Although elevated BP was perceived to be “essential” for adequate perfusion of vital organs during the early 1900s, it has been identified for decades as one of the most significant risk factors for cardiovascular disease (CVD). In a meta-analysis of 61 prospective studies, the risk of CVD increased in a log-linear fashion from SBP levels <115 to >180 mmHg and DBP levels <75 to >105 mmHg, in which the risk of death from stroke, heart disease, or other vascular disease doubles with every 20/10 mmHg increase (1, 5). A report from developing countries also implied that the burden of 47% of mortality secondary to CVDs and 44% of CVDs is attributable to high BP (6). Also the reports of the Federal Ministry of Health of Ethiopia declare that 3% of all deaths between 2005 and 2006 were the result of HTN, which accounts for the sixth top cause of death in the country (7).

Although evidence from clinical trials definitively demonstrates that antihypertensive drug therapy substantially reduces the risk of CV events and deaths in patients with high BP (8–10), the control of BP and the quality of clinical care remain generally subpar. This deficiency exacerbates the health burden on the affected populations and presents an opportunity for clinicians to enhance HTN management and care (11). The fact that uncontrolled HTN is worse in the least developed nations may indicate that there is poor hypertensive management practice and little awareness among the population about the disease. Even though the control of HTN has become a primary goal for the Federal Ministry of Health of the country in the last 10 years, it has not been implemented to its full potential (12).

Poor medication adherence, old age, obesity, high body mass index, high waist-to-hip ratio, tobacco smoking, excessive alcohol consumption, physical inactivity, and low fruit and vegetable intake were identified as factors that caused poor BP control in different clinical trials and systematic review studies (11, 13–15) but these were inconsistent across studies in Ethiopia. Therefore, this review aimed to assess BP control and the determinant factors among patients on antihypertensive medications in different regions of Ethiopia.

## Methods

### Study protocol

Identification of records, screening of titles and abstracts, and evaluation of full-text eligibility for final analysis were performed based on the Preferred Reporting Items for Systematic Reviews and Meta-Analyses (PRISMA) algorithm (16). The study protocol was registered in PROSPERO (Ref. No.: CRD42023440121).

### Data sources and search strategy

An inclusive literature search was conducted to retrieve studies that reported the prevalence of uncontrolled BP and its contributing factors in Ethiopia. We used different electronic bibliographic databases such as PubMed, Google Scholar, Hinari (Research4Life), Embase, and Scopus. Our search included studies published in English. In addition, the proceedings of professional associations and university repositories were screened. Direct Google searches and reference tracing were conducted using the bibliographies of the identified studies to include additional relevant studies omitted during the electronic database searches.

The search was conducted using the key terms from the review question. All potentially eligible studies were assessed using the following combinations of keys: prevalence, epidemiology, uncontrolled BP, HTN, uncontrolled HTN, BP, factors, determinant factors, associated factors, predisposing factors and Ethiopia. The Boolean operator terms “OR” and “AND” were used as necessary. The software Endnote 20.5 (17) was used to manage the references and remove duplicates. The search was conducted from 1 June to 29 June 2023, and all articles available online on the days of data collection were considered.

### Inclusion and exclusion criteria

Observational studies that fulfilled the following criteria were included in the final analysis: original articles published in peer-reviewed journals; articles published in English; studies that reported the prevalence of uncontrolled BP and contributing factors in any region of Ethiopia; and studies that involved hypertensive patients of any age in any healthcare setting. Qualitative studies, review articles, case reports, narrative reviews, conference abstracts with no full information, or if the authors did not respond to our inquiry on the full text, editorials, commentaries, letters to the editor, author replies, and studies that did not include quantitative data on the prevalence and contributing factors of uncontrolled BP were excluded.

### Screening and eligibility of studies

Related papers from the aforementioned databases were imported into EndNote 20.5 (17) to remove duplicates. Two independent reviewers (TK and MG), based on predefined eligibility criteria, carefully screened the selected papers based on the title, abstract, and full-text quality of each article. Deviations between the two reviewers were resolved by discussion, with the involvement of a third reviewer in selecting articles for the final review.

### Data extraction

Microsoft Excel was used for the data extraction. Two authors independently extracted data related to the study characteristics (first author, year of publication, region, study design, study population, study settings, sample size, follow-up period, and the number of patients with uncontrolled HTN).

### Quality assessment of studies

The Joanna Briggs Institute (JBI) Critical Appraisal Tool, adapted for cross-sectional (CS) studies, was used to assess the quality of the included studies. Overall, the tool has nine questions to rate the quality of the articles. The following aspects were considered to appraise the selected studies: (1) the appropriateness of the sampling frame to address the target population; (2) the appropriateness of the sampling technique for selecting the study participants and adequacy of sample size; (3) detailed description of the study subjects and settings; (4) sufficient analysis of the data and the validity and reliability of the methods used for the measurement of BP and the tools used for the classification of HTN as “controlled” and “uncontrolled”; (5) the appropriateness of the statistical analysis used; and (6) the adequacy of the response rate. Disagreements were resolved through consensus. Studies that scored five and more out of nine were considered low risk (Supplementary File S1).

### Statistical analysis

The extracted data were exported to the software STATA (version 17.0) for analysis (21). A weighted inverse variance random-effects model (18) was used to estimate the prevalence of uncontrolled HTN using the guidelines of the Seventh Joint National Committee (JNC)-7, JNC-8, and AHA/ACC as assessment tools. The variation in the pooled estimates of prevalence was adjusted through subgroup analysis according to the tools used, the regions where the studies were conducted, and the follow-up periods. Heterogeneity across the studies was assessed using the symmetry of forest plot and *I*^2^ statistics, where 25%, 50%, and 75% represent low, moderate, and high heterogeneity, respectively (19). Funnel plots and Egger regression tests were used to check for publication bias (20). A sensitivity analysis was conducted to check the stability of the summary estimate after omitting the individual studies.

## Results

### Characteristics of included studies

**Figure 1:**
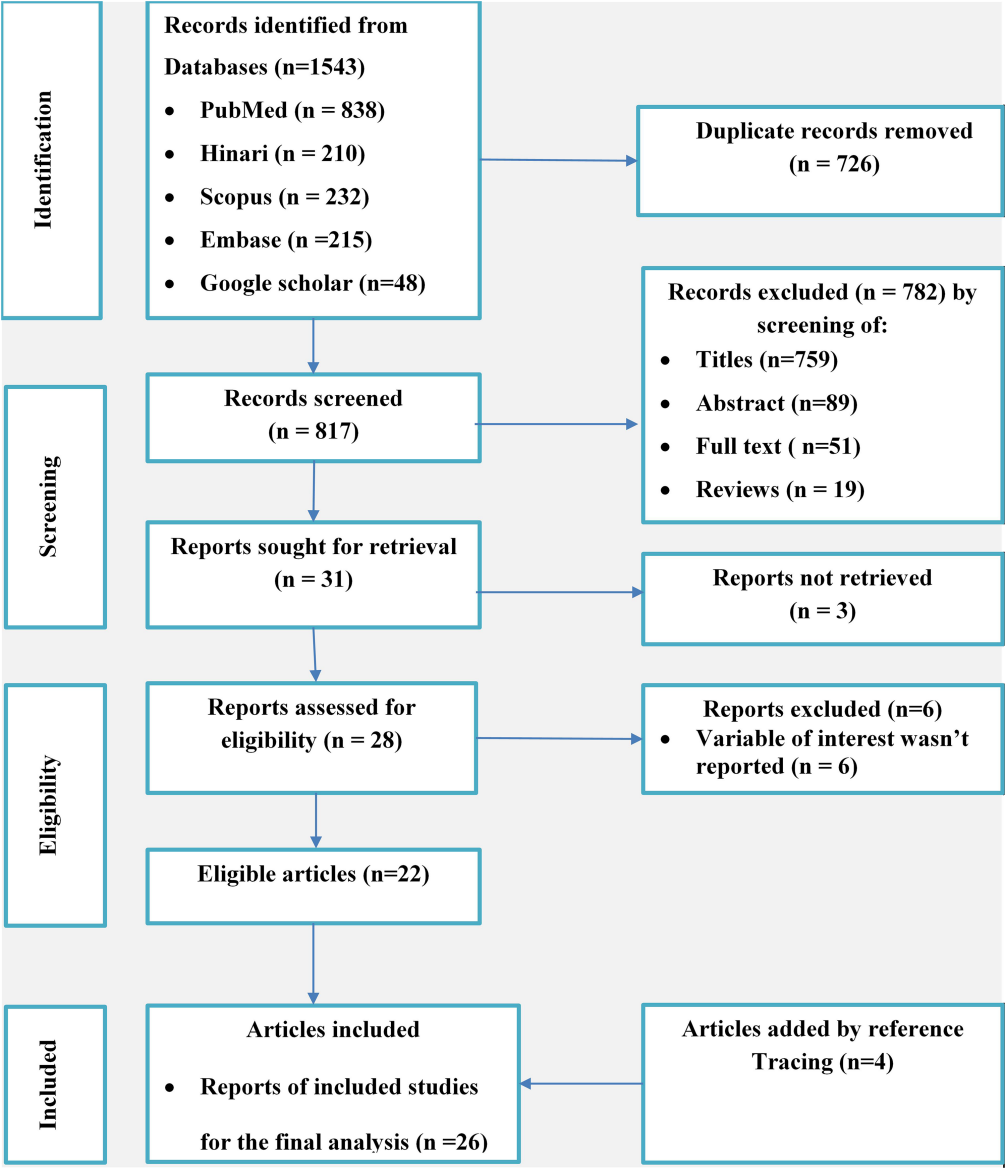
PRISMA flow diagram for the inclusion of studies in the systematic review and meta-analysis of the prevalence of uncontrolled hypertension in Ethiopia, with reasons for exclusion documented at each stage of the selection process.

A total of 1,543 potential studies were identified: 838 articles from PubMed, 210 from Hinari (Research4Life), 215 from EMBASE, 232 from Scopus, and 48 from other sources. Figure 1 shows the results of the search and the reasons for the exclusion during the study selection process. A total of 26 articles published between 2014 and 2023 were included to assess the prevalence of uncontrolled HTN, of which 11 studies (22–32) were used to assess factors contributing to uncontrolled HTN in Ethiopia. A cross-sectional, cohort, and retrospective follow-up study design were used for all the included studies. The AHA/ACC guidelines (33, 34), JNC-8 (22, 24, 25, 28, 30–32, 35–45), and JNC-7 (23, 27, 29, 46, 47) were used as assessment tools for 2, 19, and 5 studies, respectively. Eight studies were conducted in the Amhara region (22, 26, 32, 33, 35, 36, 45, 46), nine in Oromia (24, 25, 28–31, 37, 43, 47), one in the Southern Nations, Nationalities, and Peoples' Region (SNNPR) (34), four in Addis Ababa (39, 40, 42, 44), and four in Tigray (23, 27, 38, 41). In this meta-analysis 9,046 study participants were involved and 4,415 of them were found to have uncontrolled HTN. Assessment with the Joanna Briggs Institute (JBI) quality appraisal checklists indicated that none of the included studies were poor in quality and those that were poor were excluded from the meta-analysis. Table 1 presents the characteristics of the studies included.

**Table 1:** Characteristics of studies included for the systematic review and meta-analysis of the prevalence of uncontrolled HTN in Ethiopia.

Authors	Year	Region	Study design	Study population	Study setting	Sample size	NUHTN	Tool
Woldu et al.	2014	Oromia	CS	HTN patients	Hospital	288	56	JNC-7
Asgedom et al.	2016	Oromia	CS	HTN patients	Hospital	286	142	JNC-8
Abdu et al.	2017	Amhara	CS	HTN patients	Hospital	310	115	JNC-8
Berhe et al.	2017	Addis Ababa	Cohort	HTN patients	Hospitals	897	332	JNC-8
Muleta et al.	2017	Oromia	CS	HTN + DM patients	Hospital	131	74	JNC-8
Abegaz et al.	2017	Amhara	CS	HTN patients	Hospital	561	167	JNC-8
Animut et al.	2018	Amhara	Retrospective follow-up	HTN patients	Hospital	395	196	JNC-8
Yazie et al.	2018	Addis Ababa	CS	HTN patients	Hospital	356	249	JNC-8
Kebede et al.	2018	Oromia	Cohort	HTN patients	Hospitals	416	238	JNC-8
Gebremichael et al.	2018	Tigray	CS	HTN patients	Hospital	320	168	JNC-7
Abegaz et al.	2018	Amhara	CS	HTN patients	Hospital	543	62	JNC-7
Bayray et al.	2018	Tigray	CS	HTN patients	Public Offices	243	167	JNC-8
Teshome et al.	2018	Amhara	CS	HTN patients	Hospital	392	224	JNC-8
Dedefo et al.	2019	Oromia	CS	HTN + DM patients	Hospital	186	82	JNC-8
Horsa et al.	2019	Addis Ababa	CS	HTN patients	Hospital	225	166	JNC-8
Aberhe et al.	2020	Tigray	CS	HTN patients	Hospitals	391	190	JNC-7
Melaku et al.	2020	Oromia	Cohort	HTN patients	Hospital	103	62	JNC-7
Kinfe et al.	2020	Tigray	CS	HTN patients	Hospital	223	70	JNC-8
Fekadu et al.	2020	Oromia	CS	HTN patients	Hospital	297	108	JNC-8
Bogale et al.	2021	Amhara	CS	HTN patients	Hospital	203	178	AHA/ACC
Fentaw et al.	2022	Amhara	CS	HTN patients	Community	360	201	JNC-8
Sheleme et al.	2022	Oromia	CS	HTN patients	Hospital	219	123	JNC-8
Sisay et al.	2022	Addis Ababa	CS	HTN patients	Hospital	474	247	JNC-8
Yazie et al.	2022	Amhara	CS	HTN patients	Hospitals	423	202	JNC-8
Sorato et al.	2022	SNNPR	CS	HTN patients	Hospitals	406	362	AHA/ACC
Solomon et al.	2023	Oromia	CS	HTN patients	Hospitals and HC	398	234	JNC-8

SNNPR, Southern Nations, Nationalities, and Peoples region; HC, health center; NUHTN, number of uncontrolled hypertension.

### Meta-analysis

#### Prevalence of uncontrolled HTN in Ethiopia

**Figure 2:**
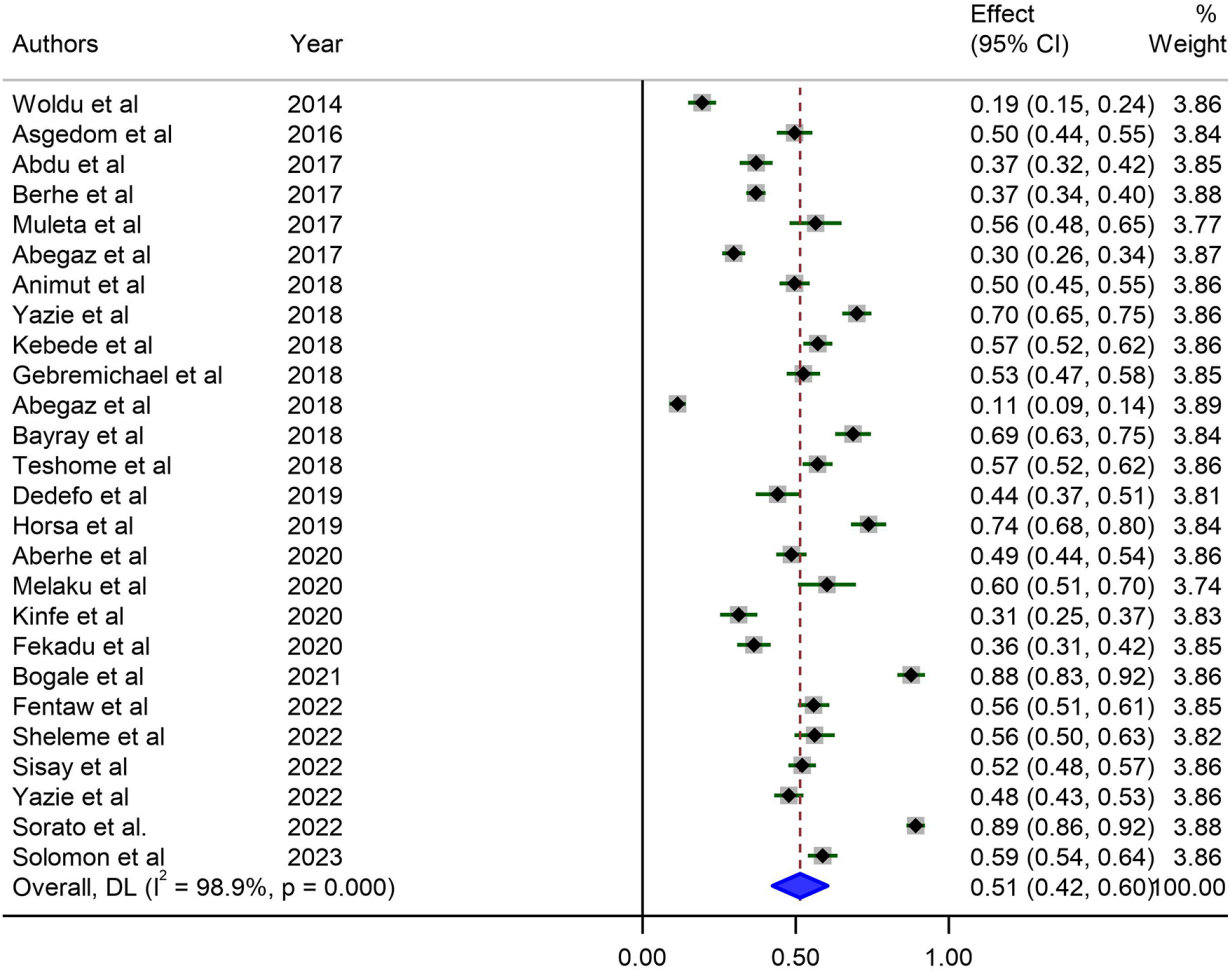
Forest plot illustrating the pooled prevalence of uncontrolled hypertension in Ethiopia from 26 observational studies. Each study's effect size (prevalence) is represented by a small solid vertical line, with its 95% CI shown by a solid horizontal line. The dashed vertical line indicates the pooled prevalence, and its 95% CI is represented by a diamond. The sizes of the shaded squares correspond to the weight assigned to each study in the pooling.

The estimated proportion of uncontrolled HTN in Ethiopia from the included 26 studies was 51% [95% confidence interval (CI), 42%–60%]. A weighted inverse variance random-effects model was used, and high degree of heterogeneity was identified between studies, as verified by the *I*^2^ statistics (*I*^2^ = 98.9%, *P* < 0.001) (Figure 2).

### Heterogeneity analysis

**Figure 3:**
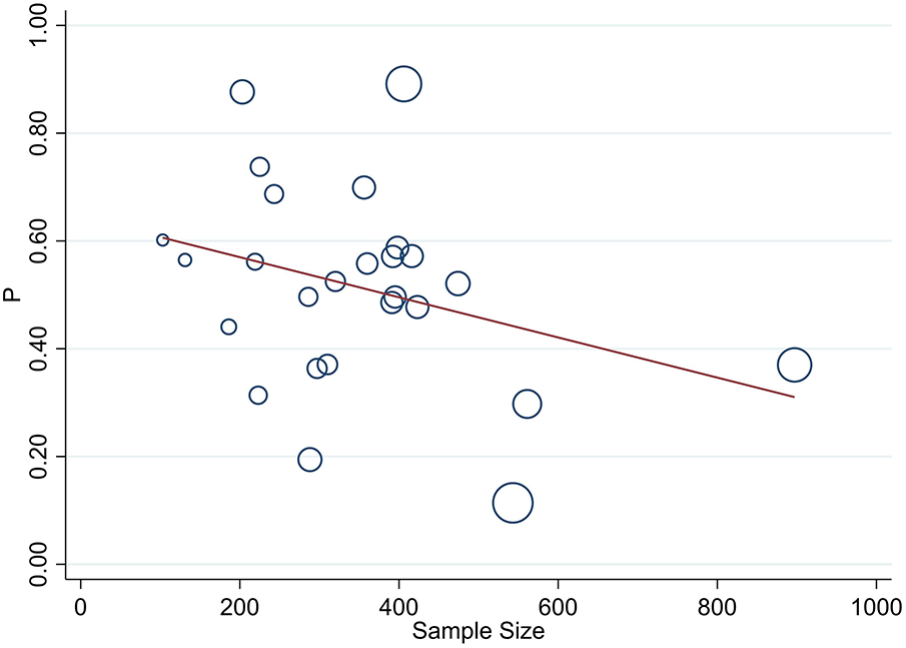
Univariate meta-regression of the prevalence of uncontrolled hypertension and sample size for analyzing heterogeneity between studies. Each circle represents a study, with the area of each circle proportional to that study's weight in the analysis. The meta-regression line is overlaid on the scatterplot, with its slope indicating the magnitude and direction of the association.

**Figure 4:**
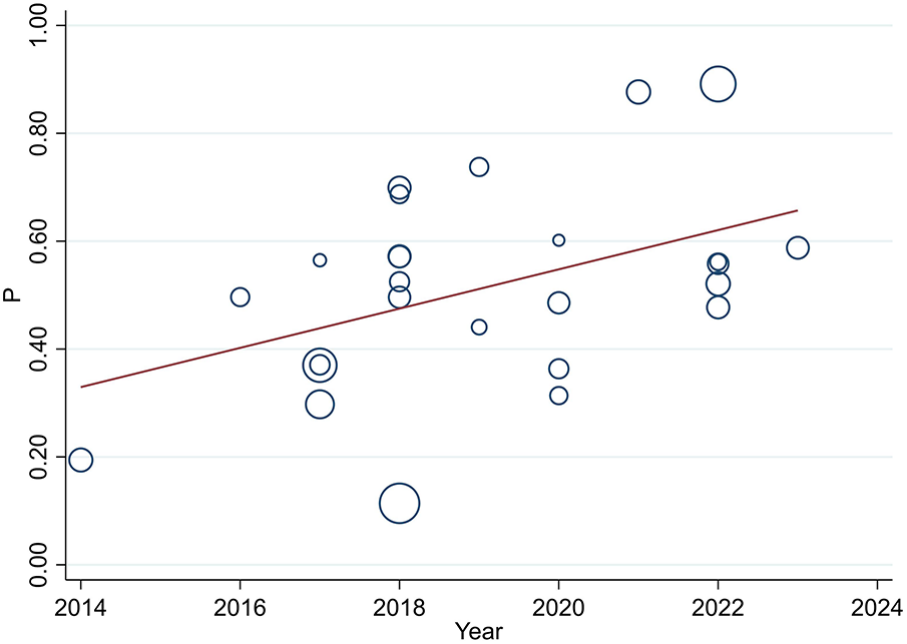
Univariate meta-regression of the prevalence of uncontrolled hypertension and year of publication for analyzing heterogeneity between studies. Each circle represents a study, with the area of each circle proportional to that study's weight in the analysis. The meta-regression line is overlaid on the scatterplot, with its slope indicating the magnitude and direction of the association.

Studies included in the analysis showed significant heterogeneity (*I*^2 ^= 98.9%; *P-*value < 0.001), which was not sufficiently treated using a weighted inverse variance random-effects model. To further analyze the source of this heterogeneity, we used a forest plot (Figure 2) as a subjective assessment and conducted subgroup analysis, sensitivity analysis, and univariate meta-regression to objectively assess the causes of heterogeneity (Table 2, Figures 3, 4).

**Table 2:** Subgroup analysis of prevalence of uncontrolled HTN in Ethiopia based on the assessment tools, regions where studies were conducted, and follow-up periods.

Groups	Subgroups	Pooled estimates (95% CI)	*I*^2^ (%)	*P*-values
Assessment tools	JNC-7	**38** (19–58)	98.8	<001
	JNC-8	**51** (45–57)	96.2	<001
	AHA/ACC	**89** (86–91)	0.0	0.594
Regions	Addis Ababa	**58** (40–76)	98.5	<001
	Amhara	**47** (29–65)	99.3	<001
	Oromia	**49** (38–59)	96.3	<001
	Tigray	**50** (36–64)	96.1	<001
Follow-up periods (months)	3	**34** (29–39)	29.5	0.234
	6	**55** (46–64)	98.5	<001
	12	**54** (44–64)	71.2	0.062

Bold values represent the pooled prevalence of uncontrolled hypertension across different subgroups.

### Subgroup analysis

Due to differences in target levels set by various tools for defining controlled and uncontrolled hypertension, along with considerations of the treatment duration before categorizing hypertension as uncontrolled, and the potential prodigious impact of cultural and lifestyle disparities among the diverse ethnic groups residing in the country, the subgroup analysis was performed based on the tools used for the classification of uncontrolled HTN, regions where studies were conducted in different parts of the country, and follow-up periods, as previously mentioned (Table 2). The highest proportion of uncontrolled HTN was observed when the AHA/ACC guidelines were used as an assessment tool (89%; 95% CI, 86%–91%), but the lowest was observed following the guidelines of the JNC7 Group 38 (95% CI, 19–58). The results of subgroup analysis based on regions where studies were conducted revealed the highest proportion of uncontrolled HTN in Addis Ababa (58%; 95% CI, 40%–76%), followed by the Tigray region (50%; 95% CI, 36%–64%). The results of subgroup analysis based on the follow-up period showed greater difference in prevalence of uncontrolled HTN in which the lowest (34%; 95% CI, 29%–39%) and highest (55%; 95% CI, 46%–64%) prevalence was found in the 3- and 6-month follow-up periods, respectively.

### Sensitivity analysis

We performed a sensitivity analysis of the prevalence of uncontrolled HTN by applying a random-effects model. Each excluded study showed a slight difference in pooled estimate of prevalence of uncontrolled HTN, the highest and lowest estimates being 53.1% (95% CI, 45.3%–60.8%) (35) and 49.99% (95% CI, 41.9%–57.9%) (34) when the respective studies were omitted.

### Meta-regression

Univariate meta-regression was used to assess sample size and publication year as the source of heterogeneity, and it revealed a slightly higher source of variation in sample size distribution when compared with the publication year (τ^2^ 0.03, *P* < 001) and (τ^2^ 0.027, *P* = 024), respectively (Figures 3, 4).

### Publication bias analysis

**Figure 5:**
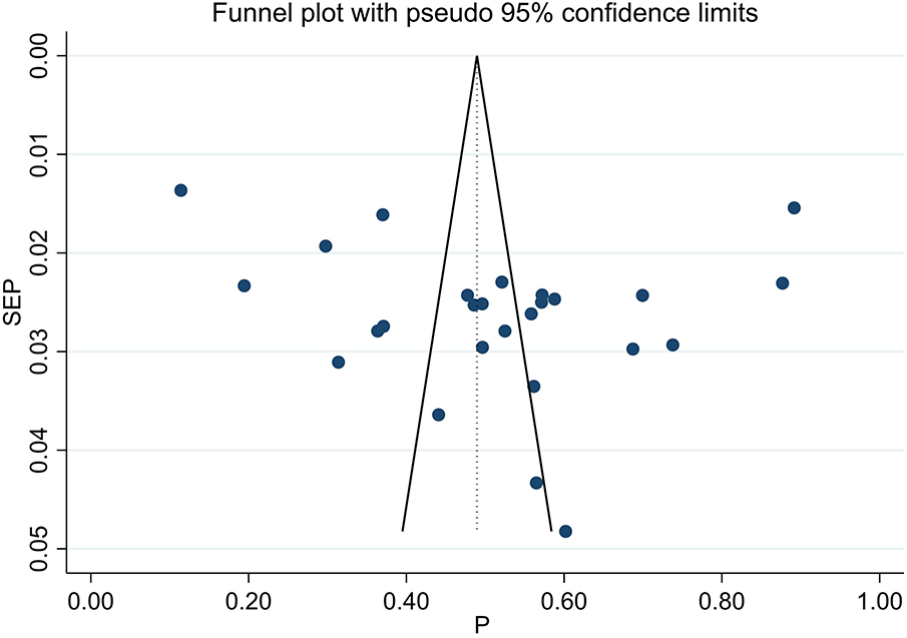
Funnel plot illustrating the prevalence of uncontrolled hypertension in Ethiopia for subjective assessment of publication bias. Each dot in the plot represents an individual study. The x-axis represents the effect size (prevalence), while the y-axis represents study precision. The vertical dotted line serves as the reference line, and the 95% confidence intervals are denoted by the distance between the two solid lines.

Publication bias was subjectively analyzed using a funnel plot (Figure 5), which was symmetrical, and we further conducted Egger's regression test, which resulted in a *P*-value of 0.274 and did not show the presence of publication bias.

### Contributing factors for uncontrolled hypertension in Ethiopia

In this meta-analysis, data regarding the effects of non-modifiable risk factors on uncontrolled HTN were estimated. The highest effect on uncontrolled HTN was observed in diabetes, followed by when the duration of the illness was longer than 5 years (Table 3).

**Table 3:** The effects of non-modifiable risk factors on uncontrolled HTN in Ethiopia.

Factors	Studies	Number of study participants	POR (95% CI)	*I*^2^ (%)	*P*-value
Age > 50 years	Aberhe et al. Muleta et al.	522	**2.47** (1.57–3.90)	0.00	0.782
Age > 60 years	Kebede et al. Sheleme et al. Dedefo et al.	821	**2.04** (0.39–10.72)	90	<0.01
Presence of comorbidity	Sheleme et al. Gebremichael et al. Melaku et al.	642	**2.51** (1.73–3.63)	14.1	0.312
DM	Abdu et al. Dedefo et al. Muleta et al.	627	**5.19** (1.41–19.11)	89.5	<0.01
Duration of illness >5 years	Dedefo et al. Muleta et al. Fekadu et al.	614	**2.82** (1.47–5.41)	0.00	0.830
Male	Fentaw et al. Fekadu et al.	657	**1.38** (1.14–1.67)	0.00	0.392

Bold values indicate the pooled effects of each contributing factor for uncontrolled hypertension.

## Discussion

This meta-analysis found that the estimated prevalence of patients with uncontrolled hypertension in Ethiopia was 51% (95% CI: 42%–60%). This eye-opening figure carries significant clinical implications, which underscores a substantial public health concern and burden, emphasizing the need for targeted interventions and improved hypertension management strategies to decrease the risk of cardiovascular diseases and associated complications, such as stroke.

This finding is in line with another meta-analysis conducted in 2020 (48); however, the analysis included an additional 13 articles and also conducted a factor analysis for the major determinants of uncontrolled HTN. The BP control rate was low and little improvement was observed in several SSA countries. In SSA, the percentage of people with controlled BP has remained low, and little progress has been made in improving BP control (49). Similarly, according to the national survey in Kenya, Ghana, and Lebanon, the prevalence of uncontrolled HTN was found to be 48.3%, 58%, and 51.1%, respectively (50–52). Global disparities in HTN control have also been reported, as there has been less improvement in low- and middle-income countries (53). However, this meta-analysis result is lower than a study conducted in Africa, which reported 79.3% of people with HTN who were receiving treatment had uncontrolled HTN (54). In addition, this result is lower than that of studies in Benin, Ghana, and Afghanistan, which reported the prevalence of uncontrolled HTN as 65.5%, 87.6%, and 77.3%, respectively. The difference could be explained by the differences in the study periods, as well as the sociodemographic and economic differences between the study populations. Moreover, this finding is lower than that of a study on the prevalence of uncontrolled HTN among people with comorbidities in SSA (55). Many patients with comorbidities may experience management with multiple medications, which could make it difficult to adhere strictly to HTN drug-taking behavior. Moreover, this problem could also be related to lifestyle modifications such as the management of HTN and drug-to-drug interactions among patients with comorbidities.

Conversely, this meta-analysis result is higher than that of a study conducted in Thailand (24.6%) (56). Our finding is substantially lower than the prevalence observed in low- and middle-income countries, India, and China, where it stands at 92.3%, 79.8%, and 91.9%, respectively (53, 57, 58). This divergence in prevalence rates underscores potential variations in hypertension control strategies, healthcare infrastructure, economic capabilities, implementations of lifestyle modifications, and patient adherence to treatment regimens across different regions. Understanding these variations is crucial for tailoring effective interventions that address the specific challenges faced by the different populations in the pursuit of optimal hypertension management across the global.

As indicated in previous studies, uncontrolled HTN increases the risk of all-cause and CVD-related morbidity and mortality (59–61). This finding also indicates that uncontrolled HTN is a major public health concern in Ethiopia. Moreover, a study showed that suboptimal BP control is responsible for a large and increasing economic and health burden in developing countries (62), and this high proportion of uncontrolled HTN could be linked with such problems.

This study revealed a significant association between patient age and uncontrolled HTN as the odds of uncontrolled HTN increased with advancing age. The findings of this study are in line with those of other studies conducted in Africa (63, 64) and elsewhere (65–69). This could be due to BP increasing as the arteries become less elastic with increasing age. This analysis further reported that sex was significantly associated with uncontrolled HTN, as men had significantly higher odds of uncontrolled HTN than women (56, 68, 70–72). This might be due to differences in health-seeking behavior, as women are better at having contact with health services than men (73). In addition, the difference could also be justified, as men are more likely to have unhealthy lifestyle practices, such as smoking, alcohol consumption, and poor dietary habits. Different studies have also revealed that the prevalence of uncontrolled HTN increases with the number of unhealthy lifestyle factors found in men (74), and adherence to recommended lifestyle modifications is common among women (75, 76).

Higher odds of uncontrolled HTN were reported in patients with diabetes mellitus (DM) and other diseases (56, 63, 65, 66, 71, 77, 78). A review of studies in SSA indicated a high burden of uncontrolled HTN among individuals with comorbidities (55). This could be explained by the fact that patients with comorbidities often require multiple medications, which could increase the risk of side effects and medication non-adherence. Previous studies have revealed that poor adherence among patients with HTN is significantly associated with uncontrolled HTN (51, 77, 79, 80). Moreover, limited access to quality healthcare services in developing countries can affect BP monitoring (55). Despite the challenges of optimal BP in patients with comorbidities, different studies have reported that patients with comorbidities are more likely to have their BP under control than those without comorbidities (81, 82). A possible explanation might be that patients with comorbidities may have increased adherence to antihypertensive medications due to fear of complications and death. Patients with comorbidities could have better awareness of the importance of controlling their BP by adhering to their medication, which could be a result of the close monitoring and improved counseling services they obtained from their healthcare providers. Although existing findings report contradictions, it is essential to prepare public health strategies to reduce the burden of uncontrolled HTN in SSA, and WHO also recommends the need for integrated care programs for the management of HTN and comorbidities (55).

Patients diagnosed with HTN for a longer duration are more likely to develop uncontrolled HTN. This finding is in line with that of other studies (51, 83, 84). This could be explained by the longer duration of HTN and greater likelihood of developing complications such as heart disease, stroke, and kidney disease. This may lead to polypharmacy, decreased adherence to therapy, and compromised lifestyle changes, leading to poor BP control.

High heterogeneity (*I*^2^ = 98.9%, *P* < 0.001) was found in this meta-analysis using a weighted inverse variance random-effects model. Univariate meta-regression identified significant negative coefficients for sample size, indicating a slight decrease in uncontrolled hypertension prevalence with larger studies, suggesting enhanced stability; and a positive one for “year of study,” suggesting a rise over time. Larger studies contribute to more reliable estimates, while the increase over time may be influenced by evolving international guidelines favoring stringent blood pressure targets for improved cardiovascular risk management. In the subgroup analysis based on assessment tools, the prevalence rates for uncontrolled HTN were found to be 38% by following the guidelines of JNC7, 51% by JNC8, and 89% by AHA/ACC. These variations could be attributed to the evolving definitions of optimal blood pressure levels in the newer international guidelines, reflecting a more conservative approach aimed at enhancing cardiovascular risk management.

## Conclusion

More than half of the patients with hypertension in Ethiopia have uncontrolled BP. Prevalent uncontrolled HTN increases the risk of cardiac, neurological, and renal complications, which may double the burden of the healthcare system in developing countries, such as Ethiopia, in addition to communicable diseases. Diabetes mellitus, advanced age, male sex, and comorbidities are among the factors that might contribute to uncontrolled HTN in Ethiopia. In light of this evidence, policymakers and healthcare professionals working in this area should implement interventional strategies and recommendations, which might be helpful in achieving optimal BP in patients with hypertension.

## Strength and limitations of the study

A comprehensive assessment was conducted to include all relevant data concerning hypertension control in patients with HTN. However, there are some limitations. The studies incorporated for the final analysis applied different standards and follow-up periods to declare uncontrolled BP. In addition to this, there is a significant heterogeneity among included studies, stemming from diverse study populations, assessment tools, and study designs.

## Data Availability

The original contributions presented in the study are included in the article/Supplementary Material, further inquiries can be directed to the corresponding author.
