# Supplementary material for: Prevalence of uncontrolled hypertension and contributing factors in Ethiopia: a systematic review and meta-analysis
Source: Front Cardiovasc Med. 2024 Apr 9;11:1335823. doi: 10.3389/fcvm.2024.1335823 (PMC11040565; doi:10.3389/fcvm.2024.1335823)
Supplement: Supplementary file 1 [file Table1.pdf]

Supplementary Table 1. Quality assessment of studies included for meta-analysis Using JBI Checklist

Author	year	Q1	Q2	Q3	Q4	Q5	Q6	Q7	Q8	Q9	Total Score	Status
Woldu et al	2014	Y	Y	Y	Y	Y	N	N	Y	Y	7/9	Included
Asgedom et al	2016	Y	Y	Y	Y	Y	N	U	Y	Y	7/9	Included
Abdu et al	2017	U	U	Y	Y	Y	N	N	Y	Y	5/9	Included
Berhe et al	2017	N	Y	Y	N	N	Y	Y	Y	Y	6/9	Included
Abegaz et al	2017	Y	Y	Y	Y	Y	Y	U	Y	NA	7/8	Included
Muleta et al	2017	N	Y	N	Y	Y	Y	N	Y	Y	6/9	Included
Abegaz et al	2018	U	Y	Y	Y	Y	Y	Y	Y	Y	8/9	Included
Animut et al	2018	Y	Y	Y	Y	Y	U	U	Y	Y	7/9	Included
Yazie et al	2018	Y	Y	Y	Y	Y	U	U	Y	Y	7/9	Included
Teshome et al	2018	Y	Y	Y	Y	Y	Y	U	Y	Y	8/9	Included
Gebremichael et al	2018	Y	Y	Y	Y	Y	Y	Y	Y	Y	9/9	Included
Bayray et al	2018	U	U	N	Y	Y	N	N	Y	Y	5/9	Included
Horsa et al	2019	Y	Y	Y	Y	N	Y	Y	N	Y	7/9	Included
Dedefo et al	2020	Y	U	N	Y	Y	Y	N	Y	Y	6/9	Included
Aberhe et al	2020	Y	Y	Y	Y	Y	N	N	Y	Y	7/9	Included
Kinfe et al	2020	Y	Y	Y	Y	Y	Y	N	Y	Y	8/9	Included
Fikadu et al	2020	Y	N	Y	Y	Y	Y	Y	Y	N	7/9	Included
Melaku et al	2020	Y	U	Y	Y	Y	Y	Y	Y	Y	8/9	Included
Kebede et al	2021	Y	Y	Y	Y	Y	U	U	Y	Y	7/9	Included
Bogale et al	2021	Y	N	N	Y	Y	Y	N	Y	Y	7/9	Included
Fentaw et al	2022	Y	Y	Y	Y	Y	Y	U	Y	Y	8/9	Included
Sisay et al	2022	Y	Y	Y	Y	Y	U	U	Y	Y	7/9	Included
Sheleme et al	2022	Y	U	U	Y	Y	Y	Y	Y	Y	7/9	Included
Yazie etal	2022	Y	Y	Y	Y	Y	Y	U	Y	Y	8/9	Included
Sorato et al.	2022	Y	Y	Y	Y	Y	U	U	Y	Y	7/9	Included
Solomon et al	2023	Y	Y	Y	Y	Y	Y	N	Y	Y	8/9	Included

Y: Yes, N:No,U:Unknown,NA:Not Applicable
